# Association of hospital safety climate and compliance with occupational safety practices among nurse interns: A cross‐sectional study using canonical correlation analysis

**DOI:** 10.1002/hsr2.70104

**Published:** 2024-10-08

**Authors:** Shan He, Zheng‐liang Li, Ying Wu, Xin Chen, Yi‐jun Chen, Wen‐feng Chen, Qian‐zhu Chen, Fang‐fang Xiong

**Affiliations:** ^1^ Department of Fundamental Nursing, School of Nursing Chongqing Medical University Chongqing China; ^2^ Department of Orthopedic Surgery The First Affiliated Hospital of Chongqing Medical University Chongqing China; ^3^ Department of Gynecology Women and Children's Hospital of Chongqing Medical University Chongqing China; ^4^ Office Jiangbei District Center for Disease Control and Prevention Chongqing China; ^5^ Department of Nursing The First Affiliated Hospital of Chongqing Medical University Chongqing China

**Keywords:** canonical correlation analysis, compliance, nurse intern, occupational health, safety climate

## Abstract

**Background and Aims:**

Nurse interns may be at a similar or higher risk than registered nurses. The key elements and mechanisms involved in the effects of safety climate on safety performance are not well understood. This study explores the relationship between the perceived hospital safety climate and compliance with occupational safety practices among nurse interns.

**Methods:**

A cross‐sectional study was conducted among 178 nurse interns in three tertiary university hospitals in Chongqing city, China. The Chinese version of the Hospital Safety Climate Scale (HSCS) was used to measure the perceived hospital safety climate of nurse interns. Compliance behavior was measured using the Compliance with Occupational Safety Practice Scale (COSPS). Canonical correlation analysis and multiple linear regression modeling were used to examine their relationship.

**Results:**

Total scores for the HSCS and COSPS were 92 (80,100) and 185 (175,185) [*M* (*P*25, *P*75)], respectively. Canonical correlation coefficients for canonical variates 1 and 2 were 0.636 (*p* < 0.001) and 0.414 (*p* < 0.001), respectively. Nurse interns' compliance with occupational safety practices was mainly influenced by management support, feedback/training, personal protective and engineering control equipment availability, and absence of job hindrance. Multiple linear regression showed that management support of HSCS accounted for 37.1% of the variance in compliance (*β* = 0.283, *p* = 0.039).

**Conclusion:**

Nurse interns reported high levels of perceived hospital safety climate and compliance with occupational safety practices. Younger nurse interns reported a lower level of perceived hospital safety climate. Nurse educators can improve interns' compliance by promoting better management support, feedback/training, personal protective and engineering control equipment availability, and fewer job hindrance.

## 1 INTRODUCTION

The concept of safety climate in industrial organizations was first introduced by Zohar in 1980, which refers to the summary of perceptions that employees share about the safety of their work environment.^1^ Hospital employee's perceptions were rarely formally assessed until 2000 when Gershon's study indicated that safety climate was an important contextual variable in the hospital environment.^2^ When safe work practices are carefully enforced at the organizational level, then employees are more likely to comply. The reason for this is that a safe hospital environment supports and reinforces individual safe behaviors, which in turn further influences employee behavior as employees influence each other. As safe behaviors are adopted throughout the organization, the pressure on noncompliant individuals will increase, motivating them to align themselves with other colleagues.^2^, ^3^

Despite the findings of researchers like Gershon, measuring safety climate from the perspective of employees in the healthcare industry are less concerned compared to studies of safety culture for patients.^4^, ^5^ Safety climate is a prerequisite for safety work practices. Increasing the safety climate will increase safety performance, which is related to patient safety and care outcomes. Recognition of the importance of safety climate has led to the development of instruments to measure safety climate in healthcare organizations, which are mostly in the form of reliability and validity scales, including Gershon's 20‐item Hospital Safety Climate Scale (HSCS),^2^ Felknor's 5‐item safety climate questionnaire,^6^ Pronovost's 10‐item safety climate scale,^7^ DeJoy's 18‐item safety climate questionnaire.^8^ Fewer studies focused on the measurement of the relationship between safety climate and outcomes in healthcare workers (HCWs). Some studies suggested that hospital safety climate was correlated with employee's compliance with safety practices and the incidence of occupational exposures.^9^, ^10^, ^11^ However, the key elements and mechanisms involved in the effects of safety climate on safety performance are not particularly well understood.

Percutaneous injuries (PCIs) are one of the major occupational injuries faced by nurses as well as interns in clinical practice. A meta‐analysis pooling 148 studies from 43 countries showed that the 1‐year prevalence of PCIs among nurses was 40.9%, second only to surgeons (72.6%) and physicians (44.5%).^12^ These injuries are largely due to needlestick and sharps injuries (NSIs). In China, a questionnaire survey of 158 hospitals in 13 provinces showed that, during three surveillance intervals, nurses all maintained the highest number of NSIs (53.90%), and students had the highest increasing rate (2.57%).^13^ NSIs are one of the major occupational injuries experienced by students and trainees in hospitals.^14^ Due to inadequate knowledge and limited clinical experience, student nurses are unable to identify potential risk factors for occupational exposure and may be at a similar or higher risk than registered nurses.^15^, ^16^ Nursing education has developed rapidly in China. In the coming years, the continuous recruitment of nursing students into schools requires the improvement of educational quality.^17^ Issues regarding the protection of preregistration nurse interns from occupational exposures require attention.

Institutionalizing a safety climate is important for the occupational safety of nurse interns. Nursing interns acquire practical nursing skills systematically through clinical practice, which is an important stage of professional development. Hospital safety climate is important for clinical interns to establish occupational safety concerns, manage occupational exposure incidents, and promote personal growth. However, the effect of safety climate on compliance with occupational safety practices among nurse interns has not been well studied yet. To gain a better understanding of hospital safety climate, especially among the nurse interns, this study aimed to (1) describe the perceived level of hospital safety climate of nurse interns, (2) describe the compliance with occupational safety practices by nurse interns, and (3) investigate the relationship between hospital safety climate and compliance.

## 2 METHODS

### 2.1 Design

The study used a cross‐sectional correlational study design and a questionnaire survey method to examine the association of perceived hospital safety climate and compliance with occupational safety practices among nurse interns.

### 2.2 Participants

The study population was enrolled nurse interns from the school of nursing, Chongqing Medical University, who were doing their clinical practicum in three affiliated hospitals of the university, and volunteered to participate in the study. The nursing school had the largest number of nursing undergraduates recruited in Chongqing. Participants were excluded if they were studying part‐time or in online learning programs. The nursing program which interns were enrolled is set up with reference to the National Standards for Quality of Teaching in Nursing of China. The Standards establishes uniform provisions for the basic requirements, practice standards, and curriculum (including clinical practice) to be met by graduates of baccalaureate nursing programs. Therefore, they are well represented in the general population of nurse interns in China.

The variable groups in the study included six subcategories of hospital safety climate and three subcategories of compliance with occupational safety practices, for a total of nine variable groups. Canonical correlation analysis (CCA) requires approximately 10 cases for each variable, so at least 90 nurse interns should participate in the study.^18^ A total of 178 nurse interns completed the electronic questionnaires and all data were used for analysis.

### 2.3 Data collection

Data were collected through an online survey platform (Wen Juan Xing, wjx.cn). To obtain cooperation, the researcher first contacted with the head nurses of the selected hospitals. Information about the research and the survey link were sent through WeChat and QQ, two web‐based social media applications. All nurse interns in the unit were asked to participate. The purpose of the study was explained at the beginning of the questionnaire. Informed consent was obtained before enrollment, and then the requirements for completing the questionnaire were explained. The time required to complete the questionnaire was approximately 10 min.

### 2.4 Ethics

This study was approved by the Institutional Review Board of the First Affiliated Hospital of Chongqing Medical University (2019‐196). All participants were assured that all data were anonymous and would be anonymous and confidential. Their participation would be completely voluntary and they could refuse or withdraw from the study at their own discretion.

### 2.5 Instruments

General information about the nurse interns was collected using a self‐report questionnaire, including gender, age, training programs, training hospital and unit.

The HSCS is a 20‐item tool developed by Gershon, to measure hospital safety climate in relation to hospital commitment to bloodborne pathogen (BBP) risk management programs.^2^ HSCS is a reliable and validated tool and was used in the United States,^19^ Japan,^20^ and China.^21^ Each item is rated on a five‐point Likert scale ranging from “strongly disagree = 1” to “strongly agree = 5,” with 3 being “neither disagree nor agree.” The HSCS has six subscales: personal protective and engineering control equipment availability (1–2), management support (3–6), absence of job hindrance (7–9), feedback/training (10–14), cleanliness/orderliness (15–17), minimal conflict/good communication (18–20). A higher total score indicates a higher level of hospital safety climate. Cronbach's alpha coefficients were 0.71–0.84 for the subscales of the original version of the HSCS. There are several Chinese translations of the HSCS, and the Nie Sheng‐xiao version was used in this study.^22^ This version has been translated and culturally adapted, localized, and tested for reliability and validity and is suitable for the Chinese nurse interns. The 20‐item Chinese HSCS had a Cronbach's alpha coefficient of 0.94. The Cronbach's alpha coefficients of the six subscales and the total score of the HSCS in this study were 0.930, 0.943, 0.902, 0.893, 0.913, 0.881, and 0.971.

Compliance behavior was measured using Compliance with Occupational Safety Practice Scale (COSPS).^23^ COSPS was developed and validated by the authors of the article. The item pool of the scale was constructed based on the theory of planned behavior, protocols of standard precautions, literature, and questionnaire survey. Through the Delphi expert consultation method, item analysis method, item‐dimension correlation coefficient, internal consistency coefficient, and factor analysis, the items of the scale were filtered, and the validity and reliability were tested. The content validity (item‐level CVI, I‐CVI) of each item of the scale ranged from 0.95 to 1.00, and the scale‐level content validity index (scale‐level CVI, S‐CVI) test showed that the experts' general agreement S‐CVI (SCVI/UA, universal agreement) was 0.93, and the average S‐CVI (S‐CVI/Ave) was 0.99. CVI (S‐ CVI/Ave) is 0.99. Three common factors were extracted from 37 items by exploratory factor analysis, namely use of protective devices, hand hygiene, and disposal and disinfection of waste. The Kaiser‐Meyer‐Olkin test coefficient was 0.949, Bartlett's sphere test showed *p* < 0.001, and the cumulative variance contribution rate was 66.706%. Each item is rated on a five‐point Likert scale from “never = 1,” “occasionally = 2,” “sometimes = 3,” “frequently = 4,” to “always = 5.” The cumulative variance contribution of the scale was 66.706%. The Cronbach's alpha coefficient was 0.968, and the 2‐week test‐retest reliability was 0.974. The Cronbach's alpha coefficients for three subscales and the total score in this study were 0.946, 0.969, 0.879, and 0.979.

### 2.6 Data analysis

Data were analyzed using IBM SPSS Statistics 27.0 (IBM Corp.). Descriptive statistics such as frequencies, percentages, and medians were used to describe the data. Mann–Whitney U test and Kruskal–Wallis test were used to examine the differences between categories. CCA was used to analyze the relationship between hospital safety climate and compliance with occupational safety practices. CCA addresses the correlation between two sets of variables, and each set of variables was analyzed as a whole when examining the linear correlation. The six dimensions of HSCS were taken as the X group variables, corresponding to the canonical variable *U*. The three dimensions of compliance with occupational safety practices were taken as the Y group variables, corresponding to the canonical variable *V*. CCA creates multiple canonical variates for these two groups of variables by linearly combining the subscale scores. First, the prerequisites for using the CCA were checked, including normality, linearity, and homoscedasticity. Sperman's correlation was then used to check for multicolinearity. A scatterplot was used to check the multivariate abnormalities for interpreting the results of the CCA. A *p *< 0.05 was considered to be statistically significant.

## 3 RESULTS

### 3.1 Demographic characteristics

Of the 178 nurse interns, 160 were female (89.89%) and 18 were male (10.11%). All interns were between 18 and 24 years old, with a mean age of (21.69 ± 0.96) years. A total of 144 (80.90%) were studying for a baccalaureate degree and 34 (19.10%) were upgrading from junior college to university. The nurse interns were placed in 29 inpatient units, including 13 surgical units (neurosurgery, orthopedics, thoracic surgery, etc.), 14 medical units (hematology, gastroenterology, nephrology, etc.) and two intensive care units.

### 3.2 Descriptive statistics of the measures

The overall HSCS score was 92 (80,100), with the maximum score of 100, indicating participants' perception of a good hospital safety climate in terms of hospital commitment to BBP risk management programs. At the item mean level, the scores of the six subscales from high to low were management support, personal protective and engineering control equipment availability, absence of job hindrance, minimal conflict/good communication, feedback/training, and cleanliness/orderliness (Table 1).

**Table 1:** Descriptive statistics of the measures [*N = 178, M (P25, P75)*].

Measure/number of items	Potential range	Range in the sample	Score
HSCS/20			
Personal protective and engineering control equipment availability/2	2–10	4–10	10 (8, 10)
Management support/4	4–20	11–20	20 (16, 20)
Absence of job hindrances/3	3–15	5–15	14 (12, 15)
Feedback/training/5	5–25	15–25	23 (20, 25)
Cleanliness/orderliness/3	3–15	3–15	14 (12, 15)
Minimal conflict/good communication/3	3–15	8–15	14 (12, 15)
Overall score	20–100	58–100	92 (80, 100)
Compliance with occupational safety practices/37			
Use of protective devices/20	20–100	66–100	100 (94, 100)
Hand hygiene/11	11–55	39–55	55 (52, 55)
Disposal and disinfection of waste/6	6–30	18–30	30 (28, 30)
Overall score	37–185	135–185	185 (175, 185)

Abbreviation: HSCS, Hospital Safety Climate Scale.

The overall COSPS score was 185 (175,185), with the maximum score of 185. At the level of item mean score, the scores of three subscales from high to low were hand hygiene, use of protective devices and disposal and disinfection of waste (Table 1). The labels for the variable groups are shown in Table 2.

**Table 2:** Labels for the variable groups of hospital safety climate and compliance with occupational safety practices.

Set	Label	Variable
Hospital safety climate	Personal protective and engineering control equipment availability	*X*_1_
	Management support	*X*_2_
	Absence of job hindrance	*X*_3_
	Feedback/training	*X*_4_
	Cleanliness/orderliness	*X*_5_
	Minimal conflict/good communication	*X*_6_
Compliance with occupational safety practices	Hand hygiene	*Y*_1_
	Use of protective devices	*Y*_2_
	Disposal and disinfection of waste	*Y*_3_

### 3.3 Differences in hospital safety climate and compliance with occupational safety practices based on demographic characteristics of participants

In terms of general information about the participants, age had a significant difference on hospital safety climate (*U* = 1895.00, *p* = 0.035). Nurse interns aged 18–22 years reported a lower level of perceived hospital safety climate than those aged 23–24 years. Age also had a significant difference on the four subscales: personal protective and engineering control equipment availability (*U* = 1964.00, *p* = 0.041), management support (*U* = 1951.00, *p* = 0.042), feedback/training (*U* = 1899.50, *p* = 0.033), and cleanliness/orderliness (*U* = 1835.00, *p* = 0.014).

For the compliance with occupational safety practices, there were no significant differences in three subscales and the total score based on demographic characteristics.

### 3.4 Correlation between sets of hospital safety climate variables and compliance with occupational safety practices

#### 3.4.1 Pearson's correlation

There were no missing data or abnormal values in the two groups of variables. By checking for the multicollinearity between variables using a correlation matrix, the correlation between hospital safety climate and compliance with occupational safety practices ranged from *r* = 0.381 to *r* = 0.854, indicating a moderate to high level of correlation (Table 3).

**Table 3:** Spearman's correlation coefficients between hospital safety climate and compliance with occupational safety practices (*N* = 178).

	Hospital safety climate						Compliance with occupational safety practices		
	*X*_1_	*X*_2_	*X*_3_	*X*_4_	*X*_5_	*X*_6_	*Y*_1_	*Y*_2_	*Y*_3_
*X*_1_	1								
*X*_2_	0.804*	1							
*X*_3_	0.776*	0.809*	1						
*X*_4_	0.704*	0.832*	0.845*	1					
*X*_5_	0.547*	0.623*	0.750*	0.802*	1				
*X*_6_	0.603*	0.629*	0.753*	0.802*	0.844*	1			
*Y*_1_	0.511*	0.555*	0.475*	0.503**	0.381*	0.415*	1		
*Y*_2_	0.523*	0.577*	0.575*	0.579*	0.421*	0.476*	0.843*	1	
*Y*_3_	0.559*	0.590*	0.525*	0.531*	0.416*	0.417*	0.762*	0.854*	1

* *p *< 0.001;

** *p *= 0.01.

#### 3.4.2 Canonical correlation

CCA showed that two of the three extracted canonical correlations were statistically significant. Wilk's λ for canonical correlation 1 was significant at 0.486 (*F* = 7.715, *p* < 0.001), indicating a moderate level of positive correlation, and for canonical correlation 2 at 0.817 (*F* = 3.607, *p* < 0.001), indicating a high level of correlation. The unstandardized linear functions were *U*_1_ (hospital safety climate) = −0.227*X*_1 _− 0.220*X*_2 _− 0.067*X*_3 _− 0.104*X*_*4*_ + 0.060*X*_5 _− 0.044*X*_6_, *V*_1_ (compliance with occupational safety practices) = −0.053*Y*_1 _‐0.050*Y*_2* *_‐0.168*Y*_3_; *U*_2_ = −0.791*X*_1_ + 0.448*X*_2 _− 0.840*X*_3 _− 0.341*X*_4_ + 0.621*X*_5 _− 0.316*X*_6_, *V*_2_ = 0.261*Y*_1 _− 0.280*Y*_2_ + 0.516*Y*_3_. The canonical correlation coefficients for canonical variates 1 and 2 were 0.636 (corresponding 40.494% overlapping variance for the first pair of canonical variates) and 0.414 (corresponding 17.128% overlapping variance for the second pair of canonical variates), respectively. That is, the first two pairs of canonical variates extracted 57.622% of the variance.

**Figure 1:**
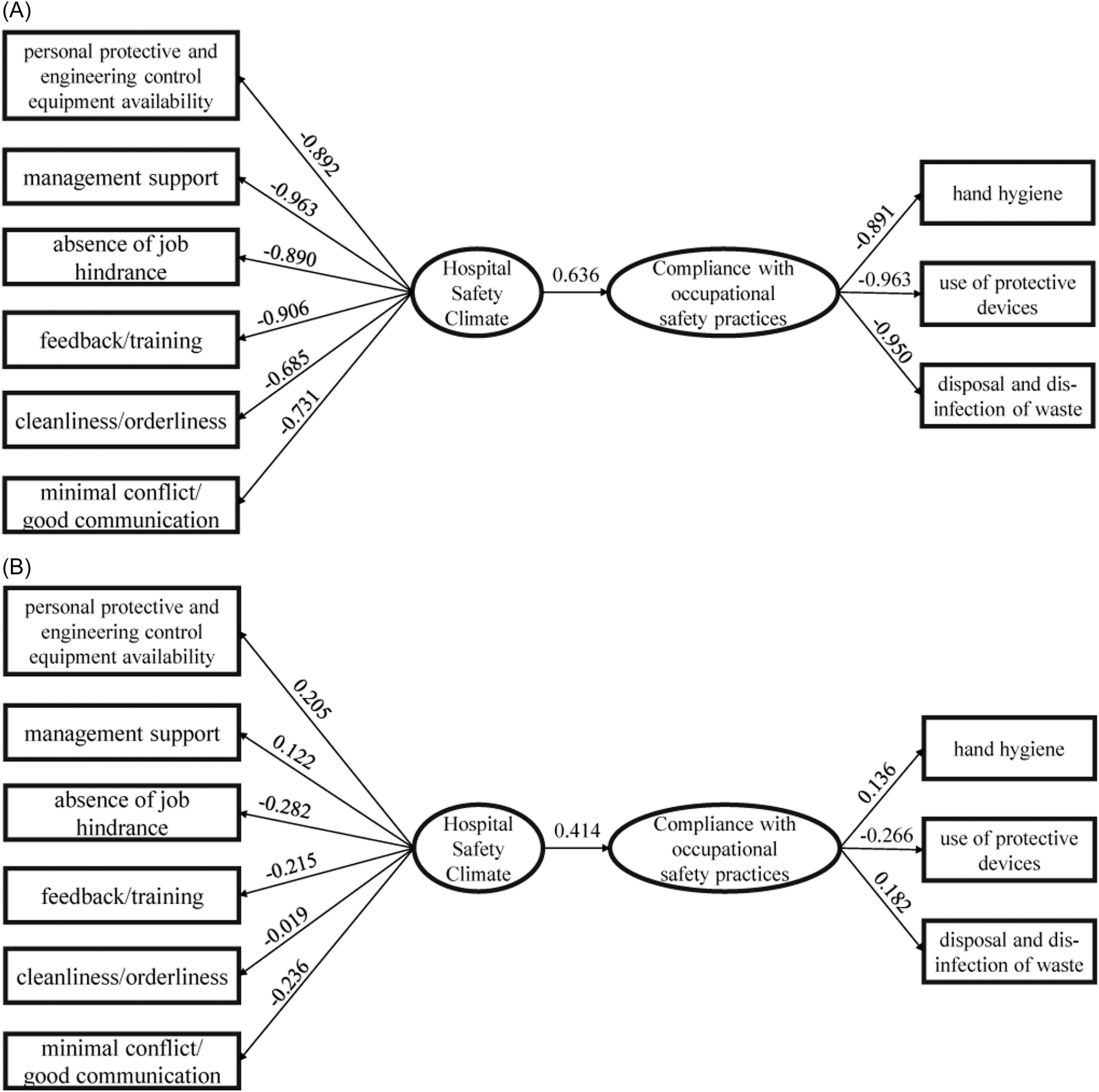
Loadings and canonical correlations for both canonical variate pairs for the data. (A) Canonical variate *U*_1_ and *V*_1_. (B) Canonical variate *U*_2_ and *V*_2_.

The loading matrices between the two pairs of canonical variates and the original variables are shown in Figure 1. Loading matrices include correlations and variables with correlations greater than 0.30 (9% of variance) are usually interpreted as part of the variate.^18^ In this study, 0.80 was taken as the cut‐off for interpreting the loadings. The first pair of canonical variates had the highest loadings on *X*_2_ (management support, −0.963), *X*_4_ (feedback/training, −0.906), *X*_1_ (personal protective and engineering control equipment availability, −0.892), and *X*_3_ (absence of job hindrance, −0.890) in the hospital safety climate set, and on *Y*_2_ (use of protective devices, −0.963), *Y*_3_ (disposal and disinfection of waste, −0.950), and *Y*_1_ (hand hygiene, −0.891) in the compliance with occupational safety practices set. Thus, higher levels of management support, feedback/training, personal protective and engineering control equipment availability, and fewer job hindrance were associated with better compliance with safety use of protective devices, proper disposal and disinfection of waste, and hand hygiene practices.

The first canonical variate pair extracted 72.318% of the variance from the hospital safety climate set and 87.457% from the compliance set. The second canonical variate pair extracted 3.974% of the variance from the hospital safety climate set and 4.071% from the compliance set. Together, the two canonical variates accounted for 76.292% of the variance in the hospital safety climate set, and 91.528% of the variance in the compliance set.

The first hospital safety climate variate accounted for 35.415% of the variance in the compliance variables. The first compliance variate accounted for 29.284% of the variance in the hospital safety climate variables.

### 3.5 Multiple linear regression analysis of hospital safety climate on compliance with occupational safety practices

In the multiple linear regression analysis, the total score of compliance with occupational safety practices was defined as the dependent variable, and gender, age and scores on the six subscales of hospital safety climate were defined as independent variables. The analysis showed that *X*_2_ (management support) accounted for 37.1% of the variance in compliance (*β* = 0.283, *p *= 0.039). This means that for every one point increase in the management support score, compliance with occupational safety practices increased by 0.283 points (Table 4).

**Table 4:** Multiple linear regression analysis of hospital safety climate on compliance with occupational safety practices (*N* = 178).

Model	Unstandardized coefficients		Standardized coefficients *β*	*t*	*p* Value
	B	SE			
Constant	90.259	22.249	—	4.057	<0.001**
Gender					
Male^a^					
Female	−0.842	2.827	−0.018	−0.298	0.766
Age, year	0.193	0.982	0.013	0.196	0.845
Hospital safety climate					
*X*_1_	1.504	1.366	0.121	1.100	0.273
*X*_2_	1.935	0.929	0.283	2.083	0.039*
*X*_3_	0.894	1.130	0.107	0.791	0.430
*X*_4_	1.031	0.798	0.199	1.291	0.198
*X*_5_	−0.805	0.934	−0.108	−0.862	0.390
*X*_6_	0.545	1.040	0.066	0.524	0.601

*Note*: *R *= 0.635, adjusted *R* ^2 ^= 0.371, *F *= 12.623 (*p *< 0.001).

^a^ Reference group.

* *p* < 0.05;

** *p* < 0.01.

## 4 DISCUSSION

This study was conducted to explore the relationship between perceived hospital safety climate and compliance with occupational safety practices among nurse interns, focusing on the characteristics within and the relationship between the variables.

Nurse interns had a positive perception of the hospital safety climate. Among six dimensions, management support, personal protective, and engineering control equipment availability scored relatively higher, while feedback and training and cleanliness/orderliness scored lower. Previous studies have measured hospital safety climate among HCWs, primarily physicians and nurses. Not much attention has been paid to student nurses. In this study, nurse interns perceived a lower level of hospital safety climate compared with the results of the survey among nurses (who were also these nurse interns' preceptors) during the same time period in the field (unpublished data). And these scores were both higher than those previously surveyed among Chinese nurses, which showed a moderate level of perceived safety climate by Qian Ping (4.00 ± 0.56),^24^ Nie Sheng‐xiao (3.91 ± 0.53),^22^ and Fang Ping‐ping (3.97 ± 0.61).^25^ One possible reason is that the contract infection measures against the coronavirus disease 19 (COVID‐19) pandemic promoted the institutional safety climate change, which included education, monitoring, and performance feedback, workplace reminders to prevent forgetfulness, the active participation of leaders and awareness of individuals to improve their practice.^26^, ^27^

Occupational exposure to bloodborne diseases remains a major risk for HCWs. Changing beliefs is the first step in promoting long‐term and sustainable changes in student behavior. Safety devices and work practice controls (e.g., caution in handling sharp devices) alone are less likely to eliminate NSIs.^28^ Institutionalizing a safety climate appears to be more economical in decreasing physical and emotional costs from injuries. Interns acquired skills by observing and imitating the behaviors of staff nurses. Therefore, safety climate influenced student nurses early in their careers. Although scores on all HSCS subscales indicated positive perceptions among nurse interns, there is room for improvement in all six hospital safety climate domains.

In this study, the 23–24‐year‐old group scored higher than the 18–22‐year‐old group on *X*_1_ (personal protective and engineering control equipment availability), *X*_2_ (management support), *X*_4_ (feedback/training), *X*_5_ (cleanliness/orderliness), and the overall HSCS score. In previous studies, individual factors such as HCW demographics (age, educational background, job tenure, work stress, etc.) and organizational factors (management decisions, organizational safety norms and expectations, safety practices, policies, and procedures) were reported to influence perceptions of the hospital safety climate in the work environment.^2^, ^4^, ^22^, ^25^ Educators should consider how younger nurse interns perceive the hospital safety climate. For nursing interns, the factors contributing to the differences in their perceived hospital safety climate may be age or age‐related psychological factors, cognitive ability, external environment, and so on. This can be further explored in future studies.

Nurse interns also had good compliance with standardized hospital infection control requirements. It should be noted that the scores of the three dimensions were quite close. In year 2019, we conducted a pilot study among nurse interns, and found that nurse interns had a high prevalence of occupational contact with BBP and a low compliance with some standard precautions. A possible reason for the change is that COVID‐19 increased HCWs' awareness of effective hand hygiene, cleaning equipment after use and appropriate personal protective equipment (PPE) use.^29^, ^30^ None of the general characteristics were found to influence on the compliance.

Standard precautions were proposed by the American CDC in 1995. It is now a widely accepted approach to preventing blood exposure to skin and mucous membranes. However, it focuses primarily on the use of barrier precautions. PPE (e.g., gloves, gowns) that provide a barrier to the shield skin and mucous membranes are easily penetrated by needles.^31^ In addition, safe devices may not be used or properly activated due to inconvenience, interruption to work and difficulty in unlearning habits that were previously considered good practice by HCWs.^32^ Only a multifaceted interventional program that combines administrative controls with safety devices and work practices will result in significant and sustained reduction in sharps injuries.^33^

The canonical correlation coefficient in this study was 0.636, which meant that students with high perception of hospital safety climate had better compliance with occupational safety practices. In other words, this study provided evidence for the need to strengthen better hospital safety climate in wards to increase their compliance by providing a more comprehensive canonical correlation between elements of hospital safety climate and compliance compared to existing studies.

It can be observed that for the first variate, nurse interns' compliance with occupational safety practices was mainly influenced by management support, feedback/training, personal protective and engineering control equipment availability, and absence of job hindrance.

The results of multiple linear regression modeling showed that the most important factor related to compliance with occupational safety practices was management support, with the adjusted *R* ^2^ being 0.371. Management's commitment, communication, and teamwork regarding safety was one of the main dimensions of the safety climate study. When senior management in healthcare organizations have a positive attitude toward improving the safety climate, the environmental equipment and safety management operations are monitored so that employees are aware of their commitment to safety.^5^ Administrations that support a strong safety climate will not only improve compliance with safe work practices, thereby reducing the risk of exposure, but will also benefit from the far‐reaching effects of the safety climate message.^2^ Hospital administrators should make it a priority to protect nurse interns from occupational blood‐borne exposures (e.g., hepatitis B, hepatitis C, AIDS, etc.) and take all reasonable measures on the wards to minimize occupational hazards. Hospitals should conduct regular occupational safety activities specifically for nurse interns, such as encouraging interns to advise on the selection of safe needles, holding occupational safety meetings, and reporting after needlestick injuries.

Preclinical preparation and adequate clinical supervision play an important role in reducing NSIs.^15^, ^34^ More frequent training in occupational safety was positively associated with improved knowledge and compliance among HCWs, as well as in nurse interns.^35^ Training should be intensified in advanced skills and techniques for the handling and disposal of needles and sharp. Standardized work habits should be developed in daily activities during students' clinical rotations. Preceptors should instruct nurse interns to avoid recapping needles, wash hands thoroughly and use PPE appropriately. Students who are uncomfortable wearing gloves should be given the opportunity to practice their technique while wearing gloves. Interns should not be allowed to perform procedures until they have reached an acceptable level of both skill and safety competency.^36^

In this study, the HSCS asked respondents whether sharps containers and disposable gloves were readily available in the work area. Both items scored higher than 4.54 points (maximum item score: 5.00), indicating the adequacy of PPE in the facility. Lack of PPE was a common factor for noncompliant behavior.^37^ And the provision of PPE such as goggles and face shields and the use of needleless intravenous systems were associated with consistent adherence among HCWs.^32^ In this study, the availability of PPE encouraged nurse interns to exhibit better adherence behaviors. In clinical settings, greater investment by administrators in safety‐engineered equipment and PPE supply is needed.

Operational barriers can affect the implementation of standard precautions by HCWs due to high workload, psychological stress, fatigue, and inadequate resources, increasing the risk of infection for patients and HCWs themselves.^38^, ^39^ The following measures can be taken to remove operational barriers to the implementation of standardized prevention: allocating appropriate working hours for interns to reduce time pressure and provide them with necessary breaks, providing mental health support services to help them cope with the stress of long working hours, and promoting teamwork and mutual support so that HCWs can work together to overcome work challenges.

The study has several limitations. First, all samples came from the three hospitals affiliated with the university; second, the cross‐sectional study design limited the ability to make causal interpretations. Future research can be conducted in different cities and multiple universities in China.

## 5 CONCLUSION

The internship period is an important period of developing nurse interns' knowledge and skills to ensure their occupational health and safety. The results of this study showed that nurse interns reported high levels of perceived hospital safety climate and compliance with occupational safety practices. Younger nurse interns reported a lower level of perceived hospital safety climate. Nurse educators can improve interns' compliance by promoting better management support, feedback/training, personal protective and engineering control equipment availability, and fewer job hindrance.

Currently, most nursing schools in China do not offer specialized courses in occupational safety and health, and there is a lack of bridging courses between theoretical learning in schools and clinical practice in hospitals. For nurse educators, it is very necessary to develop occupational protection courses to enhance interns' safety. For hospital administrators, they should consider the occupational safety of nursing interns to be as important as that of formal employees. Through policy development and management support, a good safety culture should be established in hospitals. For preceptors, they can provide specialized training workshops to address weaknesses in nurse interns' knowledge and skills based on the characteristics of the preceptorship. At the individual level, educational efforts should target nurse interns who perceived low hospital safety climate and who never report NSIs.

## AUTHOR CONTRIBUTIONS

**Shan He**: Conceptualization; data curation; software; writing—original draft. **Zheng‐liang Li**: Methodology. **Ying Wu**: Methodology; validation. **Xin Chen**: Investigation; software. **Yi‐jun Chen**: Investigation; software. **Wen‐feng Chen**: Investigation; software. **Qian‐zhu Chen**: Visualization; writing—review and editing. **Fang‐fang Xiong**: Conceptualization; writing—review and editing.

## CONFLICT OF INTEREST STATEMENT

The authors declare no conflict of interest.

## ETHICS STATEMENT

This study involves human participants. The study was approved by the Institutional Review Board of the First Affiliated Hospital of Chongqing Medical University (2019‐196). Participants gave informed consent to participate in the study before taking part.

## TRANSPARENCY STATEMENT

The lead author Shan He affirms that this manuscript is an honest, accurate, and transparent account of the study being reported; that no important aspects of the study have been omitted; and that any discrepancies from the study as planned (and, if relevant, registered) have been explained.

## Data Availability

The data that support the findings of this study are available on request from the corresponding author. The data are not publicly available due to privacy or ethical restrictions.
